# Role of VicRKX and GlnR in pH-Dependent Regulation of the *Streptococcus salivarius* 57.I Urease Operon

**DOI:** 10.1128/mSphere.00033-16

**Published:** 2016-05-18

**Authors:** Szu-Chuan Huang, Yi-Ywan M. Chen

**Affiliations:** a Department of Microbiology and Immunology and Research Center for Pathogenic Bacteria, College of Medicine, Chang Gung University, Taoyuan, Taiwan; b Graduate Institute of Biomedical Sciences, College of Medicine, Chang Gung University, Taoyuan, Taiwan; c Molecular Infectious Disease Research Center, Chang Gung Memorial Hospital, Taoyuan, Taiwan; University of Kentucky

**Keywords:** GlnR, *Streptococcus salivarius* 57.I, two-component system VicRKX, urease, pH regulation

## Abstract

Ureolysis by *Streptococcus salivarius* is critical for pH homeostasis of dental plaque and prevention of dental caries. The expression of *S. salivarius* urease is induced by acidic pH and carbohydrate excess. The differential expression is mainly controlled at the transcriptional level from the promoter 5′ to *ureI* (p_*ureI*_). Our previous study demonstrates that CodY represses p_*ureI*_ by binding to a CodY box 5′ to p_*ureI*_, and the repression is more pronounced in cells grown at pH 7 than in cells grown at pH 5.5. Recent sequence analysis revealed a putative VicR consensus and two GlnR boxes 5′ to the CodY box. The results of DNA affinity precipitation assay, electrophoretic mobility shift assay, and chromatin immunoprecipitation-PCR analysis confirmed that both GlnR and VicR interact with the predicted binding sites in p_*ureI*_. Isogenic mutant strains (*vicRKX* null and *glnR* null) and their derivatives (harboring *S. salivarius* *vicRKX* and *glnR*, respectively) were generated in a recombinant *Streptococcus gordonii* strain harboring a p*_ureI_-*chloramphenicol acetyltransferase gene fusion on *gtfG* to investigate the regulation of VicR and GlnR. The results indicated that GlnR activates, whereas VicR represses, p_*ureI*_ expression. The repression by VicR is more pronounced at pH 7, whereas GlnR is more active at pH 5.5. Furthermore, the VicR box acts as an upstream element to enhance p_*ureI*_ expression in the absence of the cognate regulator. The overall regulation by CodY, VicR, and GlnR in response to pH ensures an optimal expression of urease in *S. salivarius* when the enzyme is most needed.

**IMPORTANCE** Dental plaque rich in alkali-producing bacteria is less cariogenic, and thus, urease-producing *Streptococcus salivarius* has been considered as a therapeutic agent for dental caries control. Being one of the few ureolytic microbes in the oral cavity, *S. salivarius* strain 57.I promotes its competitiveness by mass-producing urease only at acidic growth pH. Here, we demonstrated that the downregulation of the transcription of the *ure* operon at neutral pH is controlled by a two-component system, VicRKX, whereas the upregulation at acidic pH is mediated by the global transcription regulator of nitrogen metabolism, GlnR. In the absence of VicR-mediated repression, the α subunit of RNA polymerase gains access to interact with the AT-rich sequence within the operator of VicR, leading to further activation of transcription. The overall regulation provides an advantage for *S. salivarius* to cope with the fluctuation of environmental pH, allowing it to persist in the mouth successfully.

## Abstract (precis)

Dental plaque rich in alkali-producing bacteria is less cariogenic, and thus, urease-producing *Streptococcus salivarius* has been considered as a therapeutic agent for dental caries control. Being one of the few ureolytic microbes in the oral cavity, *S. salivarius* strain 57.I promotes its competitiveness by mass-producing urease only at acidic growth pH. Here, we demonstrated that the downregulation of the transcription of the *ure* operon at neutral pH is controlled by a two-component system, VicRKX, whereas the upregulation at acidic pH is mediated by the global transcription regulator of nitrogen metabolism, GlnR. In the absence of VicR-mediated repression, the α subunit of RNA polymerase gains access to interact with the AT-rich sequence within the operator of VicR, leading to further activation of transcription. The overall regulation provides an advantage for *S. salivarius* to cope with the fluctuation of environmental pH, allowing it to persist in the mouth successfully.

## INTRODUCTION

Urease is a Ni^2+^-dependent metalloenzyme that generally consists of three subunits, α, β, and γ, encoded by *ureC*, -*B*, and -*A*, respectively (1). An exception is found in *Helicobacter pylori*, in which the α subunit (UreA) is encoded by a fusion of the *ureA* and *ureB* genes seen in other bacterial urease systems (2). The assembly of a catalytically active urease requires the products of *ureE*, -*F*, -*G*, and -*D*, known as accessory genes that encode proteins required for the incorporation of nickel into the metallocenter within the active site. Some of the bacterial urease operons contain genes encoding uptake systems for urea and Ni^2+^. For example, a H^+^-gated urea transporter is encoded by *ureI* in *H. pylori* (3). The *Streptococcus salivarius* strain 57.I *ureMQO* genes encode a Ni^2+^-specific ATP binding cassette transporter (4). Although bacterial ureases are highly conserved, the expression of bacterial urease operons is regulated by various mechanisms. For instance, *Bacillus pasteurii* and *Sporosarcina ureae* express urease constitutively, whereas the urease expression in *Proteus mirabilis* is activated by a urease-specific activator, UreR, in the presence of urea (5). On the other hand, the expression of the urease operon in *Bacillus subtilis* is regulated by GlnR, TnrA, CodY, and PucR in response to nitrogen availability (6, 7).

Urea is present abundantly in the saliva and crevicular fluid in healthy individuals (8). Thus, ureolysis by bacterial ureases to produce ammonia and CO_2_ is the primary alkali generation machinery in the oral cavity, which plays a key role in plaque pH homeostasis and dental caries prevention (9). Among the oral microflora, *S. salivarius* is the most dominant and highly ureolytic species (10). Genes encoding a functional urease are arranged as an operon in *S. salivarius* 57.I (*ureIABCEFGDMQO*) (4, 11, 12). A previous study using the chemostat culture system indicates that the expression of *S. salivarius* urease is enhanced by acidic growth pH, excess amounts of carbohydrates, and high growth rates (13). Expression analyses demonstrate that the differential expression of the urease operon in response to growth conditions is regulated mainly at the transcriptional level via a σ^70^-dependent promoter located 5′ to *ureI* (p_*ureI*_) (12). Analysis of the *cis* elements of p_*ureI*_ reveals that the 21-bp region immediately 5′ to the −35 element of p_*ureI*_ is responsible for the repression of p_*ureI*_, whereas the 40-bp region further upstream participates in the positive regulation of p_*ureI*_ (14). Furthermore, the regulation of p_*ureI*_ in response to pH is also present in the recombinant nonureolytic *Streptococcus gordonii* strain CH1, which harbors a p*_ureI_*-chloramphenicol acetyltransferase (CAT) gene (*cat*) fusion, suggesting that the expression of p_*ureI*_ is regulated by a global regulatory circuit (14). By using the chemostat culture system and various molecular analyses, we found that CodY inhibits p_*ureI*_ expression by binding to the CodY box located 2 bases 5′ to the −35 element of p_*ureI*_, and the repression is more evident during growth at pH 7 than at pH 5.5. Furthermore, in the absence of CodY, the AT tract in the CodY box also acts as an upstream (UP) element to enhance urease expression (15).

Recent sequence analysis revealed a VicR binding consensus element (5′-TGTWAH-N_5_-TGTWAH) (16, 17) and two GlnR binding consensus elements (5′-TGTNA-N_7_-TNACA) (18, 19) located 5′ to the CodY box in p_*ureI*_, raising the possibility that both VicR and GlnR participate in the urease regulatory circuit. VicR is the response regulator of the VicRKX two-component system (TCS). This system is highly conserved in low-GC-content Gram-positive bacteria (20). Different from the typical TCS, this operon also encodes a metallohydrolase, VicX, in addition to VicR and the sensor kinase VicK. VicRKX has been implicated as the master regulatory system for cell wall metabolism, cell viability, biofilm formation, genetic competence, acid stress response, and oxidative stress response (16, 21–23). This system is essential for the viability of several streptococcal species (16, 23), with the exception of *S. gordonii* CH1 (22).

GlnR, a member of the MerR family of regulators, is the key regulator for nitrogen metabolism in most Gram-positive bacteria (24). The optimal DNA binding activity of GlnR requires feedback-inhibited glutamine synthetase (FBI-GS) in *B. subtilis* (25). Generally, GlnR represses the expression of the GlnR regulon under nitrogen excess (26). A recent study by Chen and colleagues demonstrates that GlnR is activated at acidic growth pH in *Streptococcus mutans*, and the repression of the GlnR regulon at acidic pH shifts the metabolism from glutamine synthesis to ATP generation to enhance acid tolerance (19).

The regulation by VicR and GlnR of p_*ureI*_ expression under different growth conditions was investigated in this study. We found that the regulation by VicR and GlnR of p_*ureI*_ is modulated by the growth pH. GlnR activates the expression of p_*ureI*_ at acidic pH, whereas VicR represses p_*ureI*_ activity. In the absence of VicR, the AT tract within the VicR box of p_*ureI*_ acts as an UP element to further enhance p_*ureI*_ expression.

## RESULTS

### **Both VicR and GlnR bind directly to the 5′ flanking region of p*_ureI_***.

**FIG 1:**
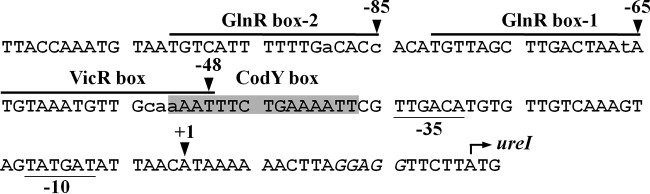
Nucleotide sequence of p_*ureI*_ and the 5′ flanking region. The transcription initiation site (+1) of the urease operon is indicated. The −10 and −35 elements of p_*ureI*_ are underlined. The translation start codon of *ureI* is indicated by a bent arrow. The ribosome binding site of *ureI* is in italics. The CodY box is shaded. The VicR box and the two GlnR boxes are overlined. Bases of the putative VicR box and GlnR boxes that are different from the consensus sequence are in lowercase.

Recent sequence analysis identified a VicR box and two putative GlnR boxes in p_*ureI*_ (Fig. 1). The VicR box, 5′-TGTAAATGTTGcaaAAT, differs by 3 bases (indicated by lowercase letters) from the consensus derived from *S. mutans* (16). The 3′ end of the VicR box overlaps the 5′ end of the CodY box by 4 bp. GlnR box 1 (5′-TGTTAGCTTGACTAAtA) and GlnR box 2 (5′-TGTCATTTTTTGaCACc) are 3 bases apart and differ from the GlnR box consensus of *S. mutans* by 1 and 2 bases (indicated by lowercase letters), respectively.

**FIG 2:**
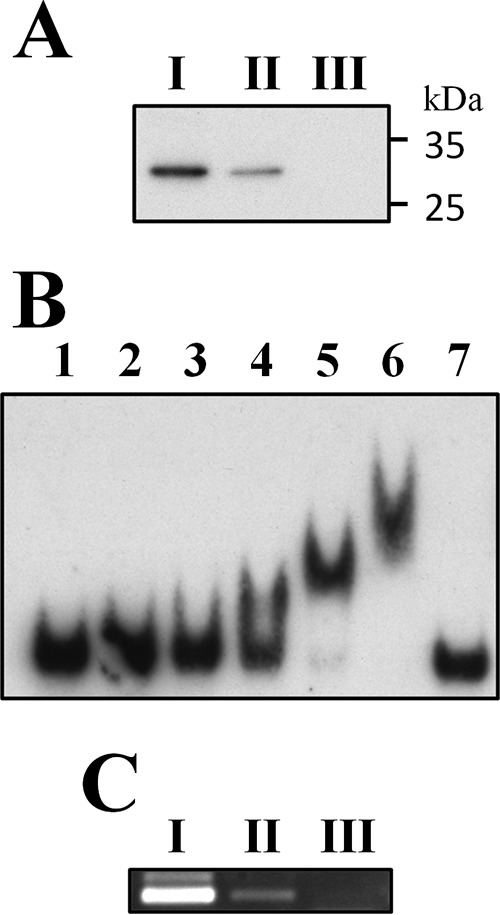
DAPA, EMSA, and ChIP-PCR analyses demonstrating *in vitro* and *in vivo* interaction of VicR with the VicR box in p_*ureI*_. (A) DAPA demonstrating specific interaction between VicR and the putative VicR box. A 100-µg amount of the total cell lysate of *S. salivarius* 57.I was used as the input control (I). Amounts of 1 mg of the total lysate were incubated with a biotin-labeled, VicR box-specific probe (an annealing product of VicR_box_S and VicR_box_AS) (II) and with a probe with mutations in the VicR box (an annealing product of mVicR_box_S and mVicR_box_AS) (III). The VicR protein in the reaction mixtures was detected by immunoblotting with anti-VicR antiserum. (B) EMSA of VicR binding to the putative VicR box in p_*ureI*_. Lane 1, probe only; lanes 2 to 6, 0.2 to 3.2 µM MalE-VicR in 2-fold increments; lane 7, 3.2 µM MalE-VicR with a specific competitor. All reactions were carried out with 0.01 pmol biotin-labeled probe. (C) The *in vivo* interaction between VicR and p_*ureI*_ was examined by ChIP-PCR assay. (I) Input control; (II) PCR product obtained from a reaction with anti-VicR antiserum; (III) final result from a reaction mixture with the preimmune rabbit serum.

A DNA affinity precipitation assay (DAPA) was performed to investigate whether the endogenous VicR interacts with p_*ureI*_. A VicR-specific signal was detected with the probe containing the putative VicR box, and the signal was abolished completely when a probe with mutations in the VicR box was used, confirming the binding specificity of VicR (Fig. 2A). As both direct and indirect interaction with the target DNA could lead to a positive result in the DAPA, an electrophoretic mobility shift assay (EMSA) was performed to verify whether VicR binds directly to the predicted VicR box in p_*ureI*_. A shift of the VicR box-specific probe was observed with 0.8 µM MalE-VicR, and a dose-dependent increase in the intensity of the signal was observed with this probe (Fig. 2B), indicating that MalE-VicR bound directly to the target. When an unlabeled probe in 300-fold excess was used in the reaction mixture, no shift was observed, confirming the binding specificity of VicR to p_*ureI*_. The recombinant MalE protein alone failed to bind to the probe (data not shown). Finally, the *in vivo* interaction between VicR and p_*ureI*_ was verified by chromatin immunoprecipitation (ChIP)-PCR assay, and the result confirmed the interaction between VicR and p_*ureI*_ (Fig. 2C).

**FIG 3:**
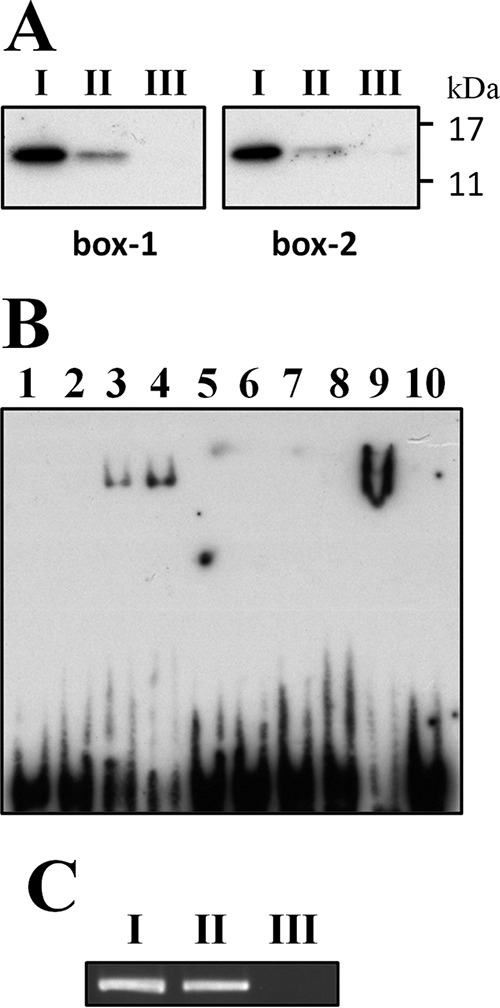
DAPA, EMSA, and ChIP-PCR analyses demonstrating *in vitro* and *in vivo* interaction of GlnR with the GlnR boxes in p_*ureI*_. (A) DAPA demonstrating specific interaction between GlnR and the putative GlnR boxes. A 1-µg amount of the total cell lysate of *S. salivarius* 57.I was used in the input control (I). Amounts of 500 µg of the total cell lysate were incubated with biotin-labeled probes specific for GlnR box 1 (an annealing product of GlnR_box-1_S and GlnR_box-1_AS) and box 2 (an annealing product of GlnR_box-2_S and GlnR_box-2_AS), respectively (II), and with probes with mutated bases in the putative GlnR boxes (annealing products of mGlnR_box-1_S plus mGlnR_box-1_AS and mGlnR_box-2_S plus mGlnR_box-2_AS) (III). The GlnR protein in the reaction mixtures was detected by immunoblotting with anti-GlnR antiserum. (B) EMSA demonstrating interaction between GlnR and the putative GlnR box 1 (lanes 1 to 5) and GlnR box 2 (lanes 6 to 10) of p_*ureI*_. Lanes 1 and 6, probe only; lanes 2 to 4 and 7 to 9, 0.2 to 0.8 µM MalE-GlnR in 2-fold increments; lanes 5 and 10, 0.8 µM MalE-GlnR with a specific competitor. All reactions were carried out with 0.01 pmol biotin-labeled probe. (C) ChIP-PCR assay demonstrating *in vivo* interaction between GlnR and p_*ureI*_. (I) Input control; (II) PCR product obtained from a reaction mixture containing anti-GlnR antiserum; (III) final result from a reaction with preimmune rabbit serum.

The same approach was used to investigate the interaction between GlnR and the putative GlnR boxes described above. Similarly, specific interactions between endogenous GlnR and the probes containing the putative GlnR box 1 and GlnR box 2, respectively, were observed in the DAPA (Fig. 3A). The binding of GlnR to the putative GlnR boxes was further confirmed by EMSA (Fig. 3B). A shift was seen with probes specific for each of the GlnR boxes, and a dose-dependent enhancement was seen with the probe specific for GlnR box 1 with increasing amounts of MalE-GlnR. Although a signal with high intensity was seen with the probe specific for GlnR box 2 in the presence of 0.8 µM MalE-GlnR, no signal was detected in the reaction mixtures with smaller amounts of MalE-GlnR. Thus, the relative degrees of affinity of GlnR for these two targets remain unclear. When an unlabeled probe in 300-fold excess was used in the reaction mixture, no shifted band was detected. As above, the recombinant MalE protein failed to interact with both probes, confirming the binding specificity of GlnR (data not shown). Notably, the addition of glutamine and glutamine synthetase to the EMSA reaction mixture did not enhance the binding (data not shown), indicating that, unlike in *B. subtilis*, FBI-GS was not required for the binding activity of *S. salivarius* GlnR. Finally, the *in vivo* interaction between GlnR and p_*ureI*_ was confirmed by the ChIP-PCR assay (Fig. 3C).

### VicRKX represses the transcription of p*_ureI_*.

**FIG 4:**
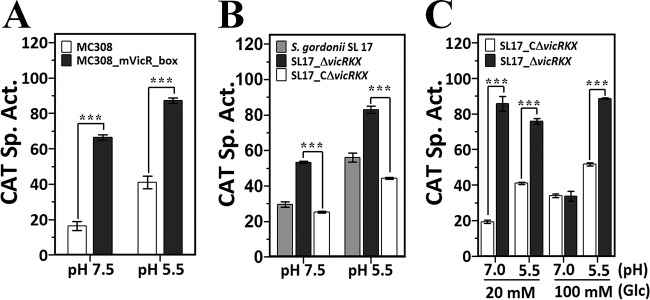
The VicRKX two-component system represses p_*ureI*_ expression. (A) Effect of the putative VicR box on p_*ureI*_ activity. The p*_ureI_*-*cat* activities in batch-grown *S. salivarius* MC308 and MC308_mVicR_box were determined by the CAT assay. Both strains were grown in BHI containing 50 mM KPO_4_ at pH 7.5 or BHI-HCl at pH 5.5. (B) Effect of VicRKX on p_*ureI*_ activity. The CAT activities in batch-grown *S. gordonii* SL17, SL17_Δ*vicRKX*, and SL17_CΔ*vicRKX* at pH 7.5 and pH 5.5 were examined. (C) Effect of growth pH and glucose concentration on p_*ureI*_ activity. The CAT activities in chemostat-grown SL17_CΔ*vicRKX* and SL17_Δ*vicRKX* were examined in cells grown at pH 7 and pH 5.5, with 20 mM and 100 mM glucose (Glc). The specific activity (Sp. Act.) was calculated as nmol Cm acetylated min^−1^ mg^−1^ total protein. Values are the mean results and standard deviations from three independent experiments. Significant differences between strains were analyzed using one-way ANOVA. ***, *P* < 0.001.

A *vicR*-deficient strain is needed to investigate whether VicR is involved in the urease regulatory circuit. However, we failed to generate a *vicR-*null recombinant strain in *S. salivarius* 57.I. Several studies have shown that VicR is essential for the viability of several oral streptococci (16, 23), and thus, it is possible that VicR plays a similar role in *S. salivarius* 57.I. To circumvent this difficulty, we first investigated the regulatory function of VicRKX in p_*ureI*_ expression by using a promoter fusion with mutations in the predicted VicR box. A derivative of *S. salivarius* strain MC308 was constructed, strain MC308_mVicR_box, in which the sequence of −64 to −59 of p_*ureI*_ (5′-TGTAAA) in the p*_ureI_*-*cat* fusion was mutated to 5′-GTCGAC. Notably, the CodY box in this fusion remains untouched. Both strain MC308 and strain MC308_mVicR_box were cultivated in brain heart infusion (BHI) at pH 7.5 and pH 5.5. A 4-fold increase in CAT activity was observed in strain MC308_mVicR_box compared to its activity in strain MC308 in cells grown at pH 7.5, whereas only a 2.1-fold increase was detected in cells grown at pH 5.5 (Fig. 4A), indicating that the putative VicR box was involved in the negative regulation of p_*ureI*_, and the repression was more evident at pH 7.5.

Since a *vicRKX*-null strain is available in *S. gordonii* CH1 (22), we examined p_*ureI*_ expression in a *vicRKX*-null derivative of *S. gordonii* strain SL17 (strain SL17_Δ*vicRKX*) and its derivative that harbors the *vicRKX* operon of *S. salivarius* 57.I (strain SL17_CΔ*vicRKX*). Notably, *S. gordonii* SL17 harbors a single copy of the p*_ureI_*-*cat* fusion on *gtfG*. In agreement with the *cis* element analysis, elevated CAT activity was detected in the *vicRKX-*null background at both pH 7.5 and pH 5.5. A wild-type level of CAT activity was observed in strain SL17_CΔ*vicRKX*, confirming that VicR repressed the transcription of p_*ureI*_ (Fig. 4B). It was also noted that the CAT activity of strain SL17_CΔ*vicRKX* was consistently lower (approximately 30%) than that of strain SL17, suggesting that *S. salivarius* VicR represses p_*ureI*_ expression more efficiently than *S. gordonii* VicR does.

To investigate the potential effects of growth pH and glucose concentration on the regulation of VicR, strains SL17_CΔ*vicRKX* and SL17_Δ*vicRKX* were grown in a chemostat, a culture system that allows tight control of the growth pH and glucose concentration. The CAT activities in cells grown at pH 7 and pH 5.5 with 20 and 100 mM glucose were examined. At pH 7, a 4.7-fold increase in CAT activity was seen in SL17_Δ*vicRKX* compared to the activity in SL17_CΔ*vicRKX* in the presence of 20 mM glucose, but comparable levels of CAT activity were detected between these two strains when cells were grown with 100 mM glucose (Fig. 4C). On the other hand, 1.8- and 1.5-fold increases in activity were seen in cells grown at pH 5.5 with 20 mM and 100 mM glucose, respectively (Fig. 4C). Collectively, the activity of VicR was modulated by both pH and carbohydrate concentration and VicRKX repressed p_*ureI*_ most effectively at neutral pH under glucose limitation.

### GlnR activates p*_ureI_* more strongly at pH 5.5.

**FIG 5:**
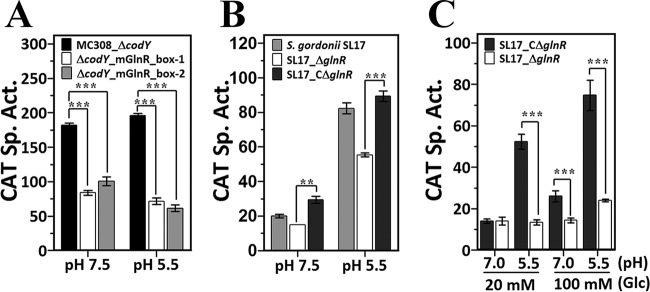
GlnR positively regulates p_*ureI*_. (A) The p*_ureI_*-*cat* activity in *S. salivarius* MC308_Δ*codY*, Δ*codY*_mGlnR_box-1, and *ΔcodY*_mGlnR_box-2. All strains were grown in BHI at pH 7.5 and pH 5.5. (B) The p*_ureI_*-*cat* activity in *S. gordonii* SL17, SL17_Δ*glnR*, and SL17_CΔ*glnR*. Cells were grown in BHI at pH 7.5 and pH 5.5. (C) Levels of p*_ureI_-cat* activity in chemostat-grown *S. gordonii* SL17_CΔ*glnR* and SL17_Δ*glnR*. The specific activities (Sp. Act.) were expressed as indicated in the legend to Fig. 4. The values are the mean results and standard deviations from three independent experiments. Significant differences between strains were analyzed using one-way ANOVA. **, *P* < 0.01; ***, *P* < 0.001.

A *glnR*-deficient strain is essential to investigate how GlnR regulates urease expression. Unfortunately, multiple attempts failed to generate a *glnR-*null mutant strain in *S. salivarius* 57.I, indicating that mutations in *glnR* are also lethal in *S. salivarius* 57.I. Thus, we initiated the study by using p*_ureI_*-*cat* fusions with mutations in the putative GlnR boxes, since the interaction between GlnR and the putative GlnR boxes 5′ to p_*ureI*_ has been confirmed (Fig. 3). Previous promoter deletion analysis indicates that a 40-bp region 22 bases 5′ to the −35 element of p_*ureI*_ exhibits a positive effect on p_*ureI*_ transcription (14). This region contains the 3′ portion of GlnR box 2 and the entire GlnR box 1, suggesting that GlnR activates p_*ureI*_ expression. As a positive effect would be more evident in a repressor-free host, i.e., strain *S. salivarius* Δ*codY*, the activity of all promoter derivatives was examined in the *codY*-deficient background. In agreement with the hypothesis, mutations in the putative GlnR boxes reduced CAT activity in batch-grown cells at both pH 7.5 and pH 5.5 and the reduction is slightly more evident at pH 5.5 (Fig. 5A), suggesting that GlnR acts as an activator for p_*ureI*_ expression and its regulatory activity on p_*ureI*_ is modulated by the growth pH.

As stated above, the nonureolytic *S. gordonii* strain seems to be a logical alternative host for studying p_*ureI*_ regulation, and luckily, a *glnR*-null *S. gordonii* derivative could be obtained. Thus, the effect of GlnR on p_*ureI*_ expression was examined in *S. gordonii* by using a similar approach as for analyzing VicR regulation. The CAT activities in the *glnR-*null *S. gordonii* SL17 (strain SL17_Δ*glnR*) and its derivative harboring the *glnR* gene of *S. salivarius* 57.I (strain SL17_CΔ*glnR*) were examined in batch-grown cells at pH 7.5 and pH 5.5. In agreement with the *cis* element analysis, the CAT activity in strain SL17_Δ*glnR* was lower than the levels in strains SL17 and SL17_CΔ*glnR* at both pH 7.5 and pH 5.5, indicating that GlnR activates p_*ureI*_ expression (Fig. 5B). It was also noticed that the CAT activity of strain SL17_CΔ*glnR* was consistently higher (approximately 40%) than that of strain SL17, suggesting that *S. salivarius* GlnR works more efficiently on p_*ureI*_ than *S. gordonii* GlnR does.

To further investigate the effects of growth pH and carbohydrate concentration on the regulation of GlnR on p_*ureI*_, strains SL17_CΔ*glnR* and SL17_Δ*glnR* were cultivated in a chemostat at pH 7 or pH 5.5 with 20 or 100 mM glucose. At pH 7, comparable expression levels were observed in strains SL17_CΔ*glnR* and SL17_Δ*glnR* under glucose limitation (20 mM), whereas a 1.8-fold increase in CAT activity was detected in strain SL17_CΔ*glnR* compared to that in strain SL17_Δ*glnR* under glucose excess (100 mM). At pH 5.5, 4-fold and 3.2-fold increases in CAT activity were seen in strain SL17_CΔ*glnR* compared to the levels in strain SL17_Δ*glnR* under 20 mM and 100 mM glucose, respectively (Fig. 5C). These results indicated that the activation by GlnR of p_*ureI*_ was modulated by both carbohydrate concentration and growth pH, and the activation was most pronounced at pH 5.5.

### The VicR box acts as a UP element of p*_ureI_*.

**FIG 6:**
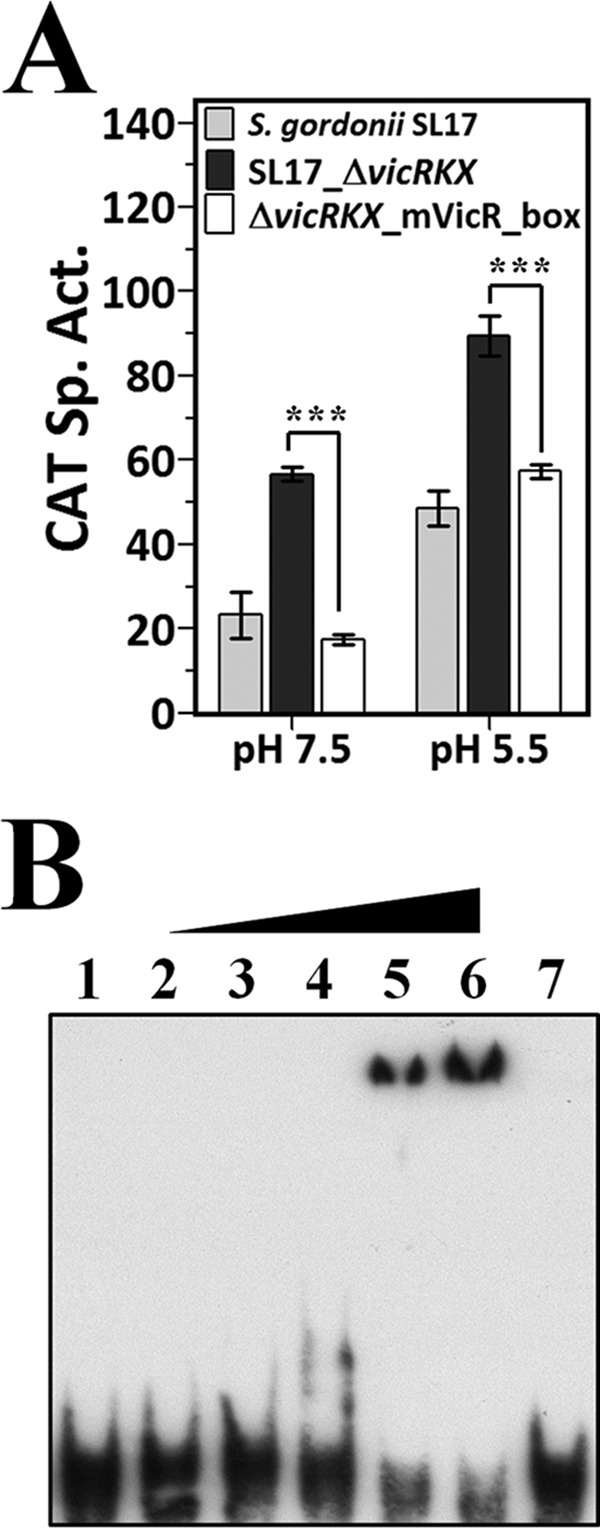
Functional analysis of the VicR box as a UP element to enhance p_*ureI*_ expression. (A) The expression of p*_ureI_-cat* in *S. gordonii* SL17, SL17_Δ*vicRKX*, and Δ*vicRKX*_mVicR_box strains was detected in cells grown in BHI at pH 7.5 and pH 5.5. The specific activity (Sp. Act.) was expressed as indicated in the legend to Fig. 4. Values are the mean results and standard deviations from three independent experiments. Significant differences between the results for strains SL17_Δ*vicRKX* and Δ*vicRKX*_mVicR_box were analyzed using one-way ANOVA. ***, *P* < 0.001. (B) EMSA of α-CTD binding to the VicR box in p_*ureI*_. Lane 1, probe only; lanes 2 to 6, 0.25 to 4 µM MalE–α-CTD in 2-fold increments; lane 7, 4 µM MalE–α-CTD with a specific competitor. All reactions were carried out with 0.01 pmol biotin-labeled probe.

As the VicR box is rich in AT, it is hypothesized that this region could act as a UP element to enhance the activity of RNA polymerase in the absence of the cognate repressor. Thus, the VicR box in the 5′ flanking region of p_*ureI*_ was mutated in strain SL17_Δ*vicRKX* to verify the possibility. Notably, the CodY box remains intact in this mutant strain. Mutations in the VicR box downregulated the CAT activity in strain SL17_Δ*vicRKX* at both pH 7.5 and pH 5.5 (Fig. 6A), suggesting that this region exhibited a positive effect on p_*ureI*_ expression in the absence of VicR. To further investigate whether this region could interact with the C-terminal domain (CTD) of the RNA polymerase α subunit (α-CTD), EMSA was carried out with a probe of 21 bp covering the entire VicR box. The result indicated that the MalE-tagged α-CTD recombinant protein interacted with the probe, and the shift was abolished when an unlabeled probe in 300-fold excess was included in the reaction mixture (Fig. 6B), confirming that the VicR box acted as a UP element to enhance p_*ureI*_ expression.

## DISCUSSION

Urease expression in both *H. pylori* and *S. salivarius* is upregulated during growth at acidic pH; however, *H. pylori* activates the expression at acidic pH by the activity of NikR and ArsR (27–29), which is different from the repression by CodY and VicR at neutral pH in *S. salivarius*. It seems most cost-effective for *H. pylori* to activate urease expression at acidic pH, as the pH of the stomach is generally below pH 5. On the other hand, the pH of oral mucosal pH is close to neutral normally (30), and therefore, *S. salivarius* gains the greatest advantage by repressing urease expression at neutral pH. The repression of *S. salivarius* p_*ureI*_ by the VicRKX system was most evident in cells cultivated at pH 7 with 20 mM glucose and less evident at pH 5.5, but surprisingly, the repressive effect was absent when cells were cultivated at pH 7 with 100 mM glucose, raising the possibility that the VicRKX system is insensitive to environmental pH under glucose excess. An attempt was made using ChIP-quantitative PCR to verify the DNA binding activity of VicR under different growth pH conditions in batch-grown *S. salivarius* 57.I. More VicR binding was detected in cells grown at pH 7.5 than at pH 5.5 (data not shown), suggesting that VicR is more active at neutral pH. Thus, the absence of repression of p_*ureI*_ by VicR at pH 7 under 100 mM glucose may result from the repression of an additional regulatory protein that exerts regulation mainly at neutral pH under glucose excess, and/or the repression by this regulatory protein is augmented in the absence of VicR under this growth condition. Our recent observations suggested that the catabolite control protein A (CcpA) also participates in the regulation of p_*ureI*_ expression. Inactivation of *ccpA* led to upregulation of p_*ureI*_ in cells grown at pH 7 with 100 mM glucose, but only marginal upregulation was seen in cells grown at pH 7 with 20 mM glucose (unpublished data). The regulation by CcpA of p_*ureI*_ is not understood currently, as no CcpA binding consensus element is found in the flanking region of p_*ureI*_. However, the effect of CcpA in response to carbohydrate concentration at pH 7 may explain the absence of upregulation in SL17_Δ*vicRKX* grown at pH 7 with 100 mM glucose.

It is intriguing that p_*ureI*_ was also repressed by the VicRKX system at pH 5.5 regardless of the glucose concentration, if neutral pH is required to activate the system. A study in *S. gordonii* indicates that inactivation of VicR reduces the tolerance of *S. gordonii* for oxidative stresses (22). Furthermore, studies in *S. mutans* have suggested that acidic growth pH could induce the oxidative stress response (31, 32). Specifically, the expression levels of genes encoding enzymes metabolizing reactive oxygen species, e.g., *sod*, *ahpC*, and *ahpF*, are upregulated in chemostat-grown *S. mutans* at pH 5 compared to their expression levels in cells grown at pH 7 (31). Thus, the activity of VicR at pH 5.5 may be part of the oxidative stress response.

The results of site-directed mutagenesis of the GlnR boxes and p_*ureI*_ expression in chemostat cultures (Fig. 5) suggest that growth pH modulates the activity of GlnR. Although it is not understood how the acidic pH activates the DNA binding activity of GlnR, the activation by acidic pH is not unique to *S. salivarius*. A recent study in *S. mutans* demonstrates that the repression of GlnR is activated at pH 5.5 (19). Thus, GlnR of oral streptococci is likely to be activated by both excess amounts of the nutrient nitrogen and acidic growth pH. Additionally, the presence of more than one GlnR box in the promoter region is also not unique to p_*ureI*_. For instance, the *glnRA* operon, the *ureABC* operon, and *tnrA* of *B. subtilis* all possess two GlnR boxes in the promoter regions (7, 18, 33), and cooperative binding of GlnR to the two GlnR boxes, 6 bp apart, has been demonstrated in the promoter of *glnRA* (33). As both GlnR box 1 and GlnR box 2 are required for GlnR-dependent activation (Fig. 5A), these two sites may participate in cooperative binding of GlnR. Furthermore, the positive regulation of GlnR in p_*ureI*_ transcription is similar to the activity of TnrA in *B. subtilis* (26). As a *tnrA* homolog is absent in most streptococcal species with known genomes (8, 34–36) and studies in *Lactococcus lactis* and *Streptococcus pneumoniae* have shown that GlnR carries out some of the functions that are exerted by TnrA in *B. subtilis* (37, 38), GlnR of *S. salivarius* is likely to function in the same way.

Studies in *S. mutans* have demonstrated that chemostat-grown cells under glucose limitation have an excess of amino acid nutrients at a high growth rate (*D* = 0.4^−1^) (39). Reduced expression of *glnRA* was observed in chemostat-grown *S. salivarius* 57.I supplemented with 20 mM glucose compared to that with 100 mM glucose (data not shown), suggesting that the nutrient nitrogen is likely to be in excess under glucose limitation. If this is true, it is expected that the activation of p_*ureI*_ transcription by GlnR should be more pronounced under glucose limitation than under glucose excess. However, we did not observe downregulation of p_*ureI*_ in strain SL17_Δ*glnR* cultivated at pH 7 under 20 mM glucose, suggesting that repressor(s) which are most active at pH 7 with limited sugar supply could mask the activation by GlnR. Based on what we have learned, the repression is likely to be governed by CodY (15) and VicR (Fig. 4B).

**FIG 7:**
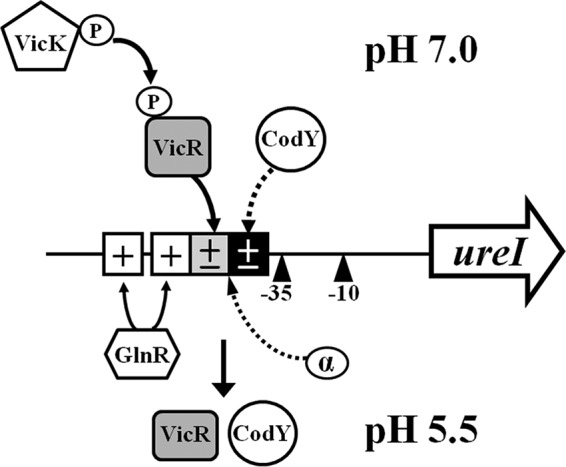
Model for urease regulation in *S. salivarius*. The relative locations of the −10 and −35 elements are indicated by triangles. The CodY box, VicR box, and GlnR boxes are indicated by black, gray, and white squares, respectively. The model suggests that CodY and VicR repress p_*ureI*_ expression at pH 7.0, whereas GlnR activates the expression at pH 5.5. In the absence of CodY and VicR, the AT tract in both the CodY box and the VicR box could act as a UP element to enhance p_*ureI*_ expression.

A working model for the regulatory network governing urease expression is proposed (Fig. 7). CodY and VicR inhibit urease expression via binding to the cognate operators in the 5′ flanking region of p_*ureI*_ at neutral pH to avoid overalkalinization of the oral cavity. GlnR activates p_*ureI*_ transcription via binding to the GlnR boxes at acidic pH to enhance acid tolerance. In the absence of the cognate repressors, both the CodY and VicR boxes could act as UP elements to enhance p_*ureI*_ expression. The complex regulation of the urease operon by these regulators links nitrogen metabolism and the acid stress response, which could ensure optimal fitness of *S. salivarius* against environmental stresses.

## MATERIALS AND METHODS

### Bacterial strains, growth conditions, and general genetic manipulations.

The bacterial strains used in this study are listed in Table 1. *S. salivarius* 57.I, *S. gordonii* CH1, and their derivatives were grown routinely in BHI (Difco) at 37°C under 10% CO_2_ atmosphere. When necessary, kanamycin (Km) was added to the culture medium at 1,000 and 250 µg · ml^−1^ for recombinant *S. salivarius* and *S. gordonii* strains, respectively. When necessary, 750 µg · ml^−1^ of spectinomycin (Sp) and 5 µg · ml^−1^ of erythromycin (Em) were used for other recombinant streptococcal strains. Recombinant *Escherichia coli* strains were maintained in L broth supplemented, where indicated, with Sp at 50 µg · ml^−1^. To obtain batch cultures grown under neutral or acidic pH conditions, cells were cultivated to mid-exponential phase (optical density at 600 nm [OD_600_] of ≈0.6) in BHI containing 50 mM KPO_4_ at pH 7.5 and in BHI that was adjusted to pH 5.5 by the addition of 2 N HCl, respectively. For continuous-culture studies, recombinant *S. gordonii* strains were grown in a Biostat B plus bioreactor (Sartorius Stedim Biotech) at a dilution rate (*D*) of 0.3 h^−1^ (generation time, 2.3 h) in medium containing 3% tryptone and 0.5% yeast extract (TY) (13). Cultures were kept at a specific growth condition for at least 10 generations to reach steady state. Furthermore, when 20 mM glucose was included in the medium, glucose was undetectable in the culture supernatant, whereas approximately 50 mM glucose remained in the culture supernatant when 100 mM glucose was used. Thus, 20 and 100 mM glucose present glucose limitation and excess, respectively.

**TABLE 1:** Bacterial strains used in this study

Strain	Resistance	Description	Reference or source
*S. salivarius* strains			
57.I		Wild-type strain	47
MC308	Sp	57.I harboring *spe-*p*_ureI_*-*cat* at *lacZ*	15
MC308_Δ*codY*	Sp, Em	MC308 *codY*::*erm*	15
MC308_mVicR_box	Sp	Nucleotides −59 to −64 of p_*ureI*_ in MC308 are mutated	This study
Δ*codY*_mGlnR_box-1	Sp, Em	GlnR box 1 of p_*ureI*_ in MC308_Δ*codY* is mutated	This study
Δ*codY*_mGlnR_box-2	Sp, Em	GlnR box 2 of p_*ureI*_ in MC308_Δ*codY* is mutated	This study
*S. gordonii* strains			
CH1		Wild-type strain	42
SL17	Sp	CH1 harboring *spe-*p*_ureI_*-*cat* at *gtfG*	This study
SL17_Δ*vicRKX*	Sp, Km	SL17 *vicR*::Ω*kan*	This study
SL17_CΔ*vicRKX*	Sp, Km, Em	SL17_Δ*vicRKX* harboring *vicRKX* (of 57.I) at *gtfG*	This study
SL17_Δ*glnR*	Sp, Em	SL17 *glnR*::*erm*	This study
SL17_CΔ*glnR*	Sp, Km	*glnR*::*erm* in SL17 is replaced by *glnR* (of 57.I)*-kan*	This study
Δ*vicRKX*_mVicR_box	Sp, Km	Nucleotides −52 to −64 of p_*ureI*_ in SL17_CΔ*vicRKX* are mutated	This study

The oligonucleotides used in this study are listed in Table 2. Synthetic DNA oligonucleotides were purchased from Genomics BioSci & Tech (Taiwan) and Integrated DNA Technologies (Singapore). Restriction enzymes and DNA-modifying enzymes were purchased from New England Biolabs (NEB). PCRs were performed with the high-fidelity DNA polymerase Blend *Taq* plus (Toyobo).

**TABLE 2:** Primers used in this study

Primer	Sequence^a^	Purpose
57.I_VicR_XhoI_S	GTAACAACTCGAGTGTATTTTACT	PCR for SL17_CΔ*vicRKX* construction
57.I_VicX_SphI_AS	GAGGAATTCCTAGCATGCCTAA	
CH1_GtfG_S	GCTAATCAAGTGACCAATG	
CH1_GtfG_BamHI_AS	CCAGTTGTTTCGGATCCTGTCTT	
CH1_GtfG_XhoI_S	GATAAGACATCTCGAGCCAATTCT	
CH1_Spec_AS	CTCTCCAAGATAACTACGAACTGCT	
CH1_VicR_S	TTTCAACCATGGAACGCTTCA	PCR for SL17_Δ*vicRKX* construction
CH1_VicR_XhoI_AS	CATCTCGAGAGCTTCACGACCATCAA	
CH1_VicR_BamHI_S	ATCGGATCCGACGACTATGTGACTAAG	
CH1_VicR_AS	TGCATATCCAGAACCTCAGA	
57.I_GlnR_NcoI_S	AGGCCATGGATGGCAAGAGAGAAAGA	PCR for SL17_CΔ*glnR* construction
57.I_GlnR_BamHI_AS	AAAGGATCCTTAGATACGCAAGTTACCAAA	
CH1_GlnR_S	GGAAATGTAACGTATATCTATCCAA	
CH1_GlnR_NcoI_AS	CATCCATGGCCTCCTTTCGTAAGATATG	
CH1_GlnA_XbaI_S	TGTTCTAGACCATAAGGAGATCACCATGCC	
CH1_GlnA2_AS	GCGAGTTCCCATGCCGCGAGATGCTG	
CH1_SGO_0212_S	CAAGATAGGATATAGAGGGTGA	PCR for SL17_Δ*glnR* construction
CH1_GlnR_XhoI_AS	TGCTCGAGTACGACGCAGTT	
CH1_GlnR_SphI_S	CTGCATGCAAGCGTAAGTATG	
CH1_GlnA1_AS	ACATGCACGTAGAACTTCATCA	
EMSA_CTD_21_S	CCTGTAAATGTTGCAAAATCC	EMSA probe for analyzing the binding of α-CTD to VicR box
EMSA_CTS_21_AS	GGATTTTGCAACATTTACAGG	
GlnR_PstI_S	AGTTCCTGCAGTTATTAGATACGCAAG	PCR for His-GlnR construction
GlnR_BamHI_AS	GAGGGGATCCATGGCAAGAGAGAAAGA	
GlnR_box 1_SalI_S	TGAGTCGACTGTAAATGTTGCAAAATTTC	Inverse PCR to mutate GlnR box 1
GlnR_box 1_SalI_AS	ACAGTCGACTCAAGC GCCGCT GTGGTGTCAA	
GlnR_box 2_NsiI_S	TTTATGCATACATGTTAGCTTGACTAATATGTAAATG	Inverse PCR to mutate GlnR box 2
GlnR_box 2_NsiI_AS	TGTATGCATAAAAAAGCTAGTTACATTTGGTAATTCT	
GlnR_box-1_S	ACCACATGTTAGCTTGACTAATATGTAAAT	DAPA and EMSA probe for analyzing the binding of GlnR to the GlnR box
GlnR_box-1_AS	ATTTACATATTAGTCAAGCTAACATGTGGT	
GlnR_box-2_S	AATGTAATGTCATTTTTTGACACCACATGT	
GlnR_box-2_AS	ACATGTGGTGTCAAAAAATGACATTACATT	
mGlnR_box-1_S	ACCACAGTCTTATGTAAAGTCATTGTAAAT	
mGlnR_box-1_AS	ATTTACAATGACTTTACATAAGACTGTGGT	
mGlnR_box-2_S	AATGTAAGTCTTATCTATCCATTGACATGT	
mGlnR_box-2_AS	ACATGTCAATGGATAGATAAGACTTACATT	
pMAL_VicR_EcoRI_S	AAGGAATTCCTAGTCATAATTCTTCATATA	PCR for MalE-VicR construction
pMAL_VicR_PstI_AS	AAGCTGCAGTGAAATCTCCTGCTTACCAGA	
pMAL_GlnR_EcoRI_S	AGGGAATTCATGGCAAGAGAGAAAGAATTAAGGCG	PCR for MalE-GlnR construction
pMAL_GlnR_PstI_AS	TTCCTGCAGTTATTAGATACGCAAGTTACCAAATTG	
pureI_4870_S	CGGACTATATTGTCAGAAACAGTC	Used in ChIP-PCR
pureI_5090_AS	CACCTAACATAAGAACCTCCTAAG	
pureI_VicR_box_SalI_S	ATAGTCGACTGTTGCAAAATTTCTGAAA	Inverse PCR to mutate VicR box in 57.I
pureI_VicR_box_SalI_AS	ACAGTCGACTATTAGTCAAGCTAACATG	
pureI_320_SalI_S	AAAGTCGACCAAATTTCTGAAAATTCGTTG	Inverse PCR to mutate VicR box in CH1
pureI_320_SmaI_AS	TTTGTCGACCCCGGGTATTAGTCAAGCTAA	
VicR_BamHI_S	GGTGGATCCATGAAAAAAATTCTAGTAGTT	PCR for His-VicR construction
VicR_PstI_AS	AAGCTGCAGTGAAATCTCCTGCTTACCAGA	
VicR_box_S	CTAATATGTAAATGTTGCAAAATTTCTGAA	DAPA and EMSA probe for analyzing the binding of VicR to the VicR box
VicR_box_AS	TTCAGAAATTTTGCAACATTTACATATTAG	
mVicR_box_S	CTAATACTAGTGTAATAATAGTATTCTGAA	
mVicR_box_AS	TTCAGAATACTATTATTACACTAGTATTAG	

a Inserted restriction recognition sites and mutated sequences are underlined.

### Purification of recombinant proteins and generation of polyclonal antisera.

The coding region of VicR was PCR amplified from *S. salivarius* 57.I using primers VicR_BamHI_S and VicR_PstI_AS and cloned into pQE30 (Qiagen) in *E. coli* M15. The identity of the recombinant plasmid was confirmed by sequencing analysis. The recombinant His-tagged VicR (His-VicR) was induced and purified under denaturing conditions using the standard methods. The identity of the recombinant protein was confirmed by matrix-assisted laser desorption ionization–time of flight (MALDI-TOF) analysis. The concentration of the purified protein was determined by Bio-Rad protein assay based on the method of Bradford (40). Using a similar approach, the coding region of GlnR was amplified from *S. salivarius* 57.I by using primers GlnR_PstI_S and GlnR_BamHI_AS, and a His-tagged GlnR protein (His-GlnR) was prepared. The purified His-VicR and His-GlnR were used to generate polyclonal antisera in rabbits by LTK BioLaboratories (Taiwan). The titers and specificities of all antisera were tested by immunoblotting.

To purify recombinant VicR and GlnR under the native condition, constructs to produce maltose binding protein-tagged VicR (MalE-VicR) and GlnR (MalE-GlnR) were generated by using pMAL-c2X (NEB). The recombinant proteins were purified by amylose affinity chromatography (NEB) and verified by MALDI-TOF analysis prior to performing the EMSA analysis.

### DAPA and Western blot analysis.

All probes used in DAPA are generated by annealing two biotin-labeled, complementary oligonucleotides. The oligonucleotides were labeled by using the Pierce biotin 3′-end DNA labeling kit. Mid-exponential phase (OD_600_ of ≈0.6) cultures of *S. salivarius* 57.I were harvested, washed once with an equal volume of 10 mM Tris (pH 7.6), and then resuspended in 1/100 of the original culture volume in the DAPA binding buffer (60 mM KCl, 10 mM Tris-HCl [pH 7.6], 5% glycerol, 0.5 mM EDTA, 1 mM dithiothreitol [DTT]). Concentrated cell suspensions were subjected to mechanical disruption in the presence of an equal volume of glass beads (0.1 mm in diameter) by homogenization in a BeadBeater (BioSpec Products) for a total of 120 s at 4°C. Amounts of 1 mg and 500 µg of the total lysate were incubated with 20 nM biotin-labeled DNA probes specific for the VicR box and for the GlnR boxes, respectively, in the DAPA binding buffer. The binding reaction was carried out under rotation at 4°C for 1 h. The DNA-protein complexes were captured by using 50 µl of streptavidin MagneSphere paramagnetic particles (Promega). The mixture was incubated at 4°C for 1 h, followed by five washes with the binding buffer. Finally, the proteins of the DNA-protein complexes were eluted in electrophoresis sample buffer, separated on 12% SDS–PAGE, and then detected by immunoblotting. Anti-VicR and anti-GlnR antisera were used at 1:2,000 and 1:20,000 dilution, respectively. The signals were detected by horseradish peroxidase-conjugated anti-rabbit IgG (GeneTex) and luminol-based reagents (Merck Millipore).

### EMSA and ChIP-PCR.

Two biotin-labeled, complementary oligonucleotides containing the target site were annealed and used in EMSA. An amount of 0.01 pmol of the annealed probe was incubated with increasing amounts of recombinant MalE-VicR, MalE-GlnR, and MalE–α-CTD in the EMSA binding buffer [50 mM Tris-HCl (pH 7.4), 50 mM KCl, 2 mM MgCl_2_, 100 ng poly(dI-dC), and 0.5 µg bovine serum albumin (BSA)]. All reactions were carried out at 4°C for 30 min, and the products were resolved on 6% nondenaturing polyacrylamide gels. Specific competition was carried out by including the same probe without labeling in a 300-fold excess. The DNA-protein complex was electrotransferred to a piece of Hybond blotting membrane (Amersham), and the signal was detected by the chemiluminescent nucleic acid detection module kit (Pierce).

ChIP-PCR assay was performed as previously described (15). The PCR was carried out by using primers pureI_4870_S and pureI_5090_AS.

### Construction of a p*_ureI_*-*cat* fusion and its derivatives in *S. gordonii* CH1 and *S. salivarius* 57.I.

The p*_ureI_*-*cat* fusion was tagged with an Sp resistance gene (*spe*) (41) and cloned into the integration vector for *S. gordonii* CH1, pMJB6 (14), which allows the integration of the promoter fusion at *gtfG*. The resulting plasmid, pSL16, was introduced into *S. gordonii* CH1 by transformation (42), and the double-crossover recombination was verified by colony PCR. The resulting strain was designated *S. gordonii* SL17.

The putative VicR box in the p*_ureI_*-*cat* fusion was mutated by site-directed mutagenesis. Briefly, the primer pairs pureI_VicR_box_SalI_S plus pureI_VicR_box_SalI_AS and pureI_320_SalI_S plus pureI_320_SmaI_AS were used in an inverse PCR using pMC300 (15) (for *S. salivarius*) and pSL16 (for *S. gordonii*) as the template, respectively. The PCR products were digested, ligated, and established in *E. coli*. The resulting plasmids were confirmed by sequencing analysis, and the correct constructs were introduced into *S. salivarius* 57.I by electroporation (12) and into *S. gordonii* Δ*vicRKX* by transformation (42). The double-crossover recombination was verified by colony PCR, and the resulting strains are designated *S. salivarius* MC308_mVicR_box and *S. gordonii* Δ*vicRKX*_mVicR_box, respectively.

The GlnR boxes in the p*_ureI_*-*cat* fusion were mutated by a similar approach. Briefly, the primer pairs GlnR_box 1_SalI_S plus GlnR_box 1_SalI_AS and GlnR_box 2_NsiI_S plus GlnR_box 2_NsiI_AS were used in PCR to introduce mutations into GlnR box 1 and 2, respectively.

### Construction of the *S. gordonii vicRKX*-deficient strain and its derivative.

All recombinant *S. gordonii* strains were generated by using PCR ligation mutagenesis (43). To inactivate *vicRKX* in *S. gordonii* SL17, the 5′ and 3′ flanking fragments of *vicR* were generated from *S. gordonii* CH1 by using the primer pairs CH1_VicR_S plus CH1_VicR_XhoI_AS and CH1_VicR_BamHI_S plus CH1_VicR_AS, respectively. The PCR products were digested and then ligated to the 5′ and 3′ ends of an Ω*kan* (44) fragment. The ligation mixture was used to transform *S. gordonii* SL17, with selection for Km resistance. The double-crossover recombination was verified by colony PCR, and the resulting strain was designated SL17_Δ*vicRKX*. The region encoding the 41st to 92nd amino acids of VicR in strain SL17_Δ*vicRKX* was replaced by Ω*kan*.

To generate a *vicRKX* recombinant strain, two fragments for integrating the *vicRKX* gene of *S. salivarius* at *gtfG* were generated from SL17_Δ*vicRKX* by primer pairs CH1_GtfG_S plus CH1_GtfG_BamHI_AS and CH1_GtfG_XhoI_S plus CH1_Spec_AS, respectively. A DNA fragment containing the *vicRKX* operon was amplified from *S. salivarius* 57.I by PCR using primers 57I_VicR_XhoI_S and 57.I_VicX_SphI_AS. A DNA fragment containing the nonpolar Em resistance gene (*erm*) from Tn*916*ΔE (45), which does not possess a promoter or a transcription terminator, was also prepared by PCR. All PCR products were digested and used in a ligation reaction. The ligation mixture was used to transform strain SL17_Δ*vicRKX*, and the allelic exchange event in the Em-resistant transformants was verified by colony PCR. The resulting strain, SL17_CΔ*vicRKX*, harbors a copy of *erm*-tagged *vicRKX* on *gtfG*.

### Construction of the *S. gordonii glnR*-deficient and derivative strains.

The *glnR* gene of *S. gordonii* CH1 was inactivated by the method described above. Briefly, the 5′ and 3′ flanking fragments of *glnR* were generated from *S. gordonii* CH1 by primer pairs CH1_SGO_0212_S plus CH1_GlnR_XhoI_AS and CH1_GlnR_SphI_S plus CH1_GlnA1_AS, respectively. These two fragments were ligated to the 5′ and 3′ ends of a nonpolar *erm* fragment, and the ligation mixture was used to transform *S. gordonii* SL17. The double-crossover recombination was verified by colony PCR, and the resulting strain was designed strain SL17_Δ*glnR.* The region encoding the 12th to 76th amino acids of GlnR was replaced by a nonpolar *erm* in this strain*.*

An *S. gordonii* *glnR*-derived strain was generated as described above. Briefly, the 5′ and 3′ flanking fragments were generated from SL17_Δ*glnR* by PCR with the primer pairs CH1_GlnR_S plus CH1_GlnR_NcoI_AS and CH1_GlnA_XbaI_S plus CH1_GlnA2_AS, respectively. A DNA fragment containing *glnR* was generated from *S. salivarius* 57.I by PCR using primers 57I_GlnR_NcoI_S and 57.I_GlnR_BamHI_AS. A ligation mixture of all three fragments and a DNA fragment containing a nonpolar *kan* cassette (44) that lacks a promoter and a transcription terminator was prepared and used to transform strain SL17_Δ*glnR*. The ligation was prepared to favor the formation of a construct comprising the 5′ flanking fragment followed by *glnR* of *S. salivarius*, the *kan* fragment, and the 3′ flanking fragment. The correct allelic exchange event in the Km-resistant transformants was verified by colony PCR, and the resulting strain was designed SL17_CΔ*glnR*.

### CAT assay.

Total protein lysates from the concentrated cell suspensions were subjected to mechanical disruption as described previously (14). The CAT activities were determined by the method of Shaw (46), and the specific activities were calculated as nmol of Cm acetylated min^−1^ mg^−1^.

### Statistical analysis.

Statistical analysis was performed using one-way analysis of variance (ANOVA) with GraphPad Prism (version 5) software.
